# Genome-wide identification of target genes for miR-204 and miR-211 identifies their proliferation stimulatory role in breast cancer cells

**DOI:** 10.1038/srep25287

**Published:** 2016-04-28

**Authors:** Hyunkyung Lee, Seungyeon Lee, Hansol Bae, Han-Sung Kang, Sun Jung Kim

**Affiliations:** 1 Department of Life Science, Dongguk University-Seoul, Goyang, Republic of Korea; 2 Research Institute and Hospital, National Cancer Center, Goyang, Republic of Korea

## Abstract

MiR-204 and miR-211 (miR-204/211) share the same seed site sequence, targeting many of the same genes. Their role in cancer development remains controversial, as both cell proliferative and suppressive effects have been identified. This study aimed to address the relationship between the two structurally similar microRNAs (miRs) by examining their target genes in depth as well as to reveal their contribution in breast cancer cells. Genome-wide pathway analysis with the dysregulated genes after overexpression of either of the two miRs in MCF-7 breast cancer cell identified the “Cancer”- and “Cell signaling”-related pathway as the top pathway for miR-204 and miR-211, respectively. The majority of the target genes for both miRs notably comprised ones that have been characterized to drive cells anti-tumorigenic. Accordingly, the miRs induced the proliferation of MCF-7 and MDA-MB-231 cells, judged by cell proliferation as well as colony forming assay. Tumor suppressors, MX1 and TXNIP, were proven to be direct targets of the miRs. In addition, a high association was observed between miR-204 and miR-211 expression in breast cancer tissue. Our results indicate that miR-204/211 serve to increase cell proliferation at least in MCF-7 and MDA-MB-231 breast cancer cells by downregulating tumor suppressor genes.

MicroRNAs (miRs) are short non-coding RNAs of 21–23 nucleotides in length, which are synthesized as longer precursors and processed in the cytosol to produce the mature form^1^. Various chromosomal regions have been mapped as the miR locus, including RNA coding regions, introns, and intergenic spacers. At present, over 2,500 mature miRs have been identified in human^2^. Several cellular functions are controlled by miRs: cell development, cell signaling, tumorigenesis and metastasis, and cell cycle^3^,^4^,^5^. MiRs that are dysregulated in cancer are of special interest, because in many cases, they are responsible for oncogenesis through targeting and downregulating cancer-related genes. For example, miR-21 is upregulated in a few cancers, and it targets tumor suppressors such as PDCD4^6^, PTEN^7^, and TPM1^8^ to drive tumor development. Meanwhile, miR-145 is downregulated in certain cancers, including colon cancer^9^, breast cancer^10^, and lung cancer^11^, and it targets oncogenes such as MYC, MUC1, and OCT4 to limit cancer development. Interestingly, a few miRs show double-edge sword-like activity in cancer; that is, they behave either as oncogenic or tumor-suppressive depending on the cancer type or cellular condition. An example is miR-23b, which is highly expressed in glioma wherein it deteriorates VHL, contributing to tumor development^12^. However, the expression of miR is suppressed in bladder cancer, reactivating the tumor suppressors^13^. miR-7 is another example showing the double activity depending on tumor tissue. In renal cell carcinoma, miR-7 is upregulated, and it promotes cell migration and proliferation^14^, whereas it works as a tumor-suppressive miR in breast cancer^15^. MiR-375 behaves in an even more intricate way. It shows completely opposite activities in the same prostate cancer tissue; that is, it is oncogenic in 22Rv1 cancer cells yet tumor-suppressive in PC-3 cancer cells^16^.

In breast cancer, various miRs have been identified to act at different cancer stages and in different cell types^17^. MiR-17 is an example possessing dual activity, suppressing cell proliferation by targeting AIB1^18^ while inhibiting a tumor suppressor, HBP1, in MCF-7^19^.

MiR-204 and miR-211 (miR-204/211) have similar nucleotide sequences in the mature form, as they share the same seed sequence and have only two different nucleotides in the whole sequence. However, they are encoded from different chromosomal loci (miR-204 on 9q21.12 and miR-211 on 15q13.3). From each chromosomal site containing the miR, TRPM3 and TRPM1 are encoded respectively, explaining the high structural similarity of the two miRs. This high homology makes the two miRs target almost the same gene sets, and therefore, they drive cells in the same way, either oncogenic or tumor-suppressive. Both oncogenic and tumor-suppressive activities have been shown for miR-204/211. First, the exogenous expression of miR-211 in a colorectal cancer cell line (HCT-116) induced higher levels of cell proliferation, tumor growth, and cell migration and downregulated CHD5 (a tumor suppressor)^20^. Second, in prostate cancer, miR-204 acted as a tumor suppressor in two prostatic adenocarcinoma cell lines (LNCaP and 22Rv1) and as an oncomiR in two neuroendocrine-like cancer cell lines (PC-3 and CL1)^21^ and in three metastatic carcinoma cell lines, DU145^22^, VCaP, and H660^23^. Tumor-suppressive activity of miR-204 and/or miR-211 has been also found in other cancer cell lines such as hepatoma (HepG2)^24^, esophageal cancer (EC109 and TE10)^25^, and gastric cancer (AGS)^26^.

In breast cancer, miR-204 is downregulated and plays a tumor-suppressive role. The decreased expression of miR is associated with poor prognosis in breast cancer patients^27^. The STAT3/BCL-2/surviving pathway^28^ and AKT/mTOR/Rac1 signaling pathway^29^ are involved in the course of apoptosis induced by miR-204 and cell invasion caused by the genomic loss of miR-204. MiR-211 in breast cancer acts as a tumor suppressor in some cases, downregulating Runx2^30^, or as an oncomiR in other cases, promoting the growth of MCF-7 cancer cells^31^.

In this study, to elucidate the role of miR-204/211 in breast cancer cells, a genome-wide expression alteration and a pathway annotation analysis were carried out after inducing upregulation and downregulation in MCF-7 and MDA-MB-231 cells. The cellular behavioral change, including cell proliferation and colony formation, was also examined.

## Results

### MiR-204/211 affect genes related with “cellular growth and proliferation” and “cell death”

**Figure 1:**
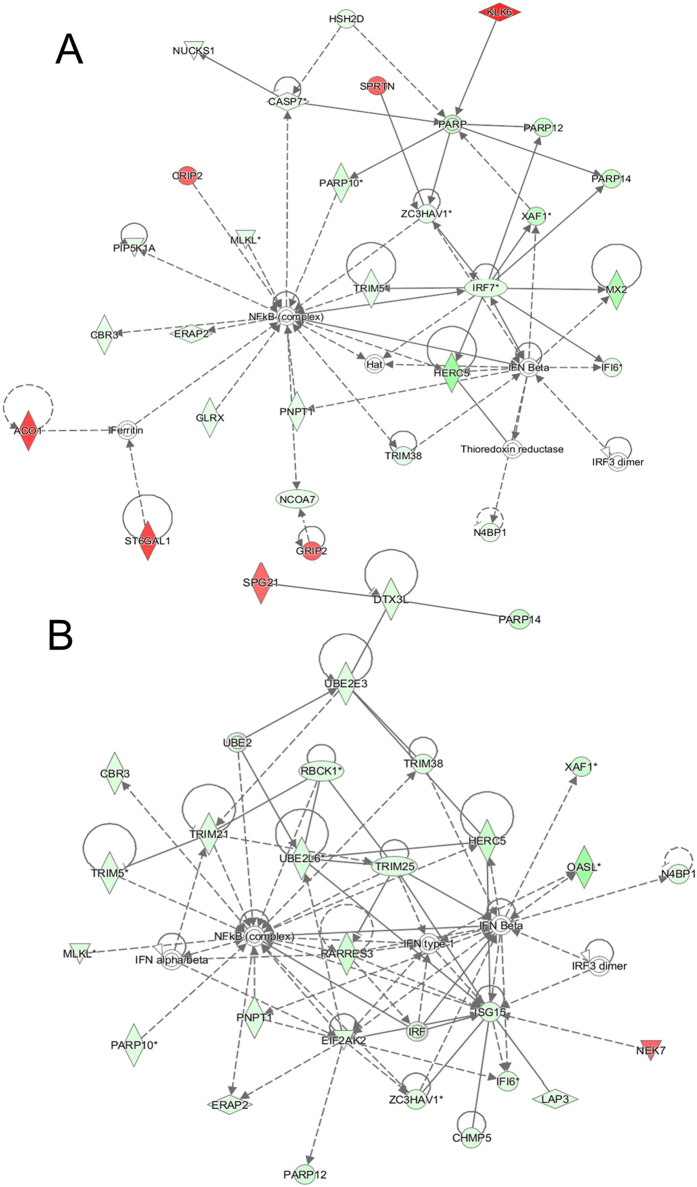
Highest confidence network of genes displaying altered expression by miR-204/211 in MCF-7. The highest confidence network was constructed using IPA from 453 and 384 dysregulated genes by miR-204 (**A**) and miR-211 (**B**), respectively. The top network was “Infectious Disease, Auditory Disease, and Cancer” for miR-204 and “Infectious Disease, Cell Signaling, Cell-To-Cell Signaling, and Interaction” for miR-211. Upregulated genes are shaded in red, while downregulated genes are shaded in green, with the color intensity signifying the magnitude of expression change. Solid and dashed lines represent direct and indirect interactions, respectively. Networks for miR-204/211 in MCF-10A are given in Supplementary Fig. S2.

**Figure 2:**
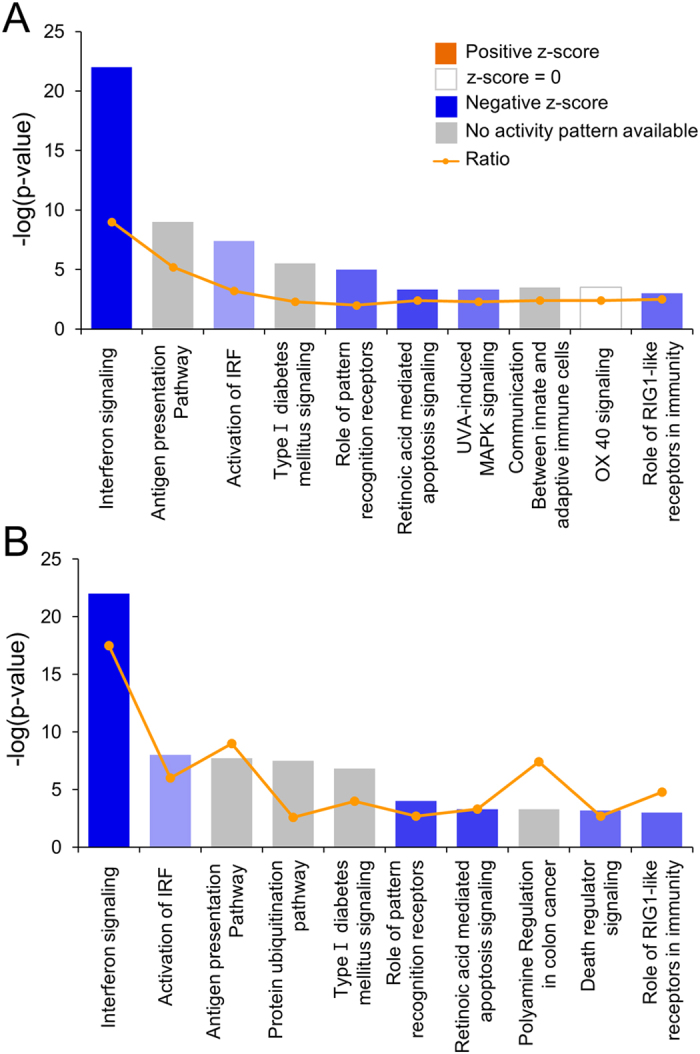
Pathways most strongly associated with the genes significantly dysregulated by miR-204/211 in MCF-7. The top 10 functional categories are given for altered genes by miR-204 (**A**) and miR-211 (**B**) overexpressed in MCF-7 cells. In both miRs, “Interferon Signaling” appeared as the top pathway having the lowest *p*-value. The IPA software assigns a *p*-value based on the likelihood of obtaining the observed number of pathway-related molecules in a given dataset by chance alone. The threshold line denotes the *p* = 0.05 level. The line graph represents the ratio of affected genes to the total number of genes in a pathway. The top 10 functional categories by miR-204/211 in MCF-10A are given in Supplementary Fig. S3.

To identify target genes regulated by miR-204/211 and the relevant pathways at a genome-wide level, each miR was overexpressed in mammary gland-originated cells. To do this, the miR mimics were transiently transfected into MCF-10A (normal cells) and MCF-7 (cancer cells). The overexpression of the miRs was confirmed by RT-PCR (Supplementary Fig. S1), and then cellular RNAs were analyzed on expression microarrays. A pool of genes satisfying our statistical criteria and showing an expression change higher than 1.5 fold was filtered, and this resulted in 145 and 453 genes for miR-204 and 162 and 384 genes for miR-211 in MCF-10A and MCF-7, respectively. To identify the most significant gene network, an Ingenuity Pathway Analysis (IPA) was carried out for the genes. The top network in MCF-10A for both miR-204/211 was “Cancer, Cellular Movement, Organismal Injury, and Abnormalities” (Supplementary Figs S2 and S3). The top networks in MCF-7 for miR-204/211 were “Infectious Disease, Auditory Disease, and Cancer” and “Infectious Disease, Cell Signaling, Cell-To-Cell Signaling, and Interaction,” respectively (Fig. 1). In both cases of MCF-7, “Interferon Signaling” was identified as the top canonical pathway (Fig. 2).

**Figure 3:**
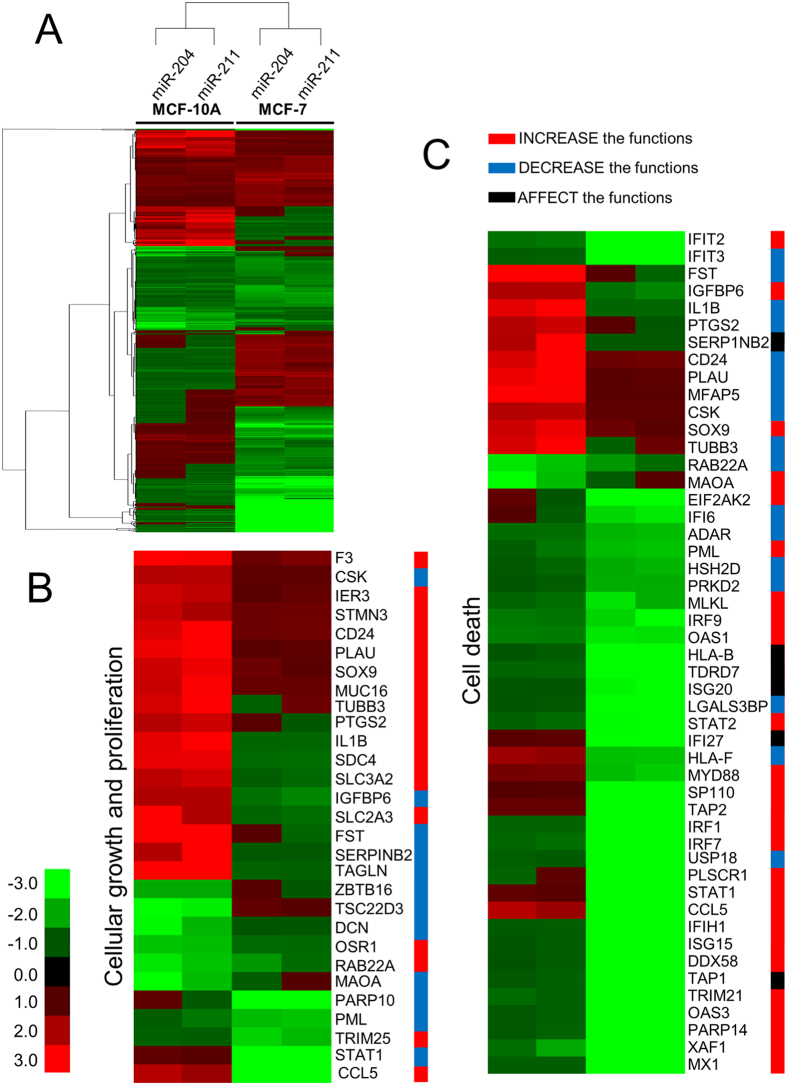
Hierarchical clustering of genes affected by miR-204/211 in MCF-10A and MCF-7. (**A**) The heatmap was constructed with genes dysregulated by miR-204 or miR-211. Each column represents gene expression change by the indicated miR overexpressed in MCF-10A or MCF-7. If a gene satisfies the criteria of differential expression (≥1.5), at least in one of the four samples, it is adopted for comparison. The genes with the green color are “downregulated,” whereas the red color signifies “upregulated” compared to those in mock miR-overexpressing cells. Of the genes that appeared in panel (**A**), the ones related to “cellular growth and proliferation” (**B**) and “cell death” (**C**) were clustered in separate heatmaps. The colored bar to the right of the gene symbol signifies the activity of the cellular biofunctions based on information from Qiagen (http://www.sabiosciences.com/pathwaysonline/). Genes in red and blue bar indicate that their expression is changed to directions to increase and decrease the functions, respectively. Genes in black bar are to affect the function.

The clustering statistical analysis method was applied over the gene pool to gain insight into the relationship between the target genes of miR-204/211. Interestingly, the closeness of the regulated gene clustering depended on the cell type rather than the miR (Fig. 3A). In practice, the gene sets affected by miR-204/211 in MCF-10A were grouped closely as with those in MCF-7. This fact implies that the two miRs share common regulatory routes at a high ratio and that their effect in cancer cells is different from that in normal cells.

**Figure 4:**
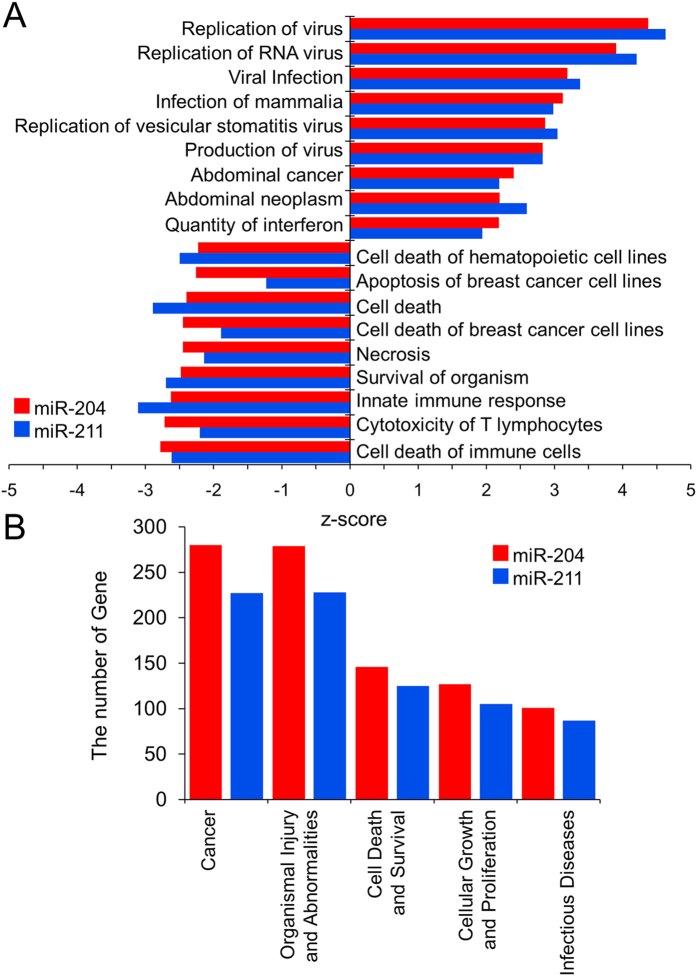
Significant diseases and functions associated with differentially expressed genes by miR-204/211 overexpressed in MCF-7. Significant diseases and functions are deduced using IPA from the dysregulated genes by miR-204/211 overexpressed in MCF-7. (**A**) Bars with positive z-scores indicate that functional activity is increased, whereas negative z-scores mean decreased activity. (|z-score| >2.0, *p*-value < 0.05) (**B**) Top five diseases and functions are shown in terms of the affected gene number. The number of molecules in the graph was determined by IPA, and the result suggests that the greatest number of differentially expressed genes is associated with cancer.

To elucidate the role of miR-204/211 in cellular activity, especially cell survival and death, genes involved in these categories were screened, and their expression change was observed. Notably, many genes responsible for cellular growth and differentiation as well as cell death were dysregulated toward a direction to stimulate proliferation and suppress cell death, resulting in a potential increase of cell survival (Fig. 3B,C). For example, F3 on the top of Fig. 3B is known to increase the “cellular growth and proliferation” effect when upregulated (therefore denoted in red bar)^32^. In fact, the gene was upregulated by both miR-204 and miR-211, especially in MCF-10A. In this way, we found many genes such as IER3, STMN3, and CD24 were upregulated by the miRs. On the other hand, CSK on the second top in Fig. 3B is known to decrease the activity when upregulated (therefore denoted in blue bar) and upregulated in our microarray assay. In addition, cancer- or neoplasm-related genes were upregulated by both the miRs, while apoptosis- or necrosis-related genes was downregulated (Fig. 4). These results prompted us to examine the role of miR-204/211 in determining the cell fate at the cell level.

### MiR-204/211 stimulate proliferation of MCF-10A, MCF-7, and MDA-MB-231 cell

**Figure 5:**
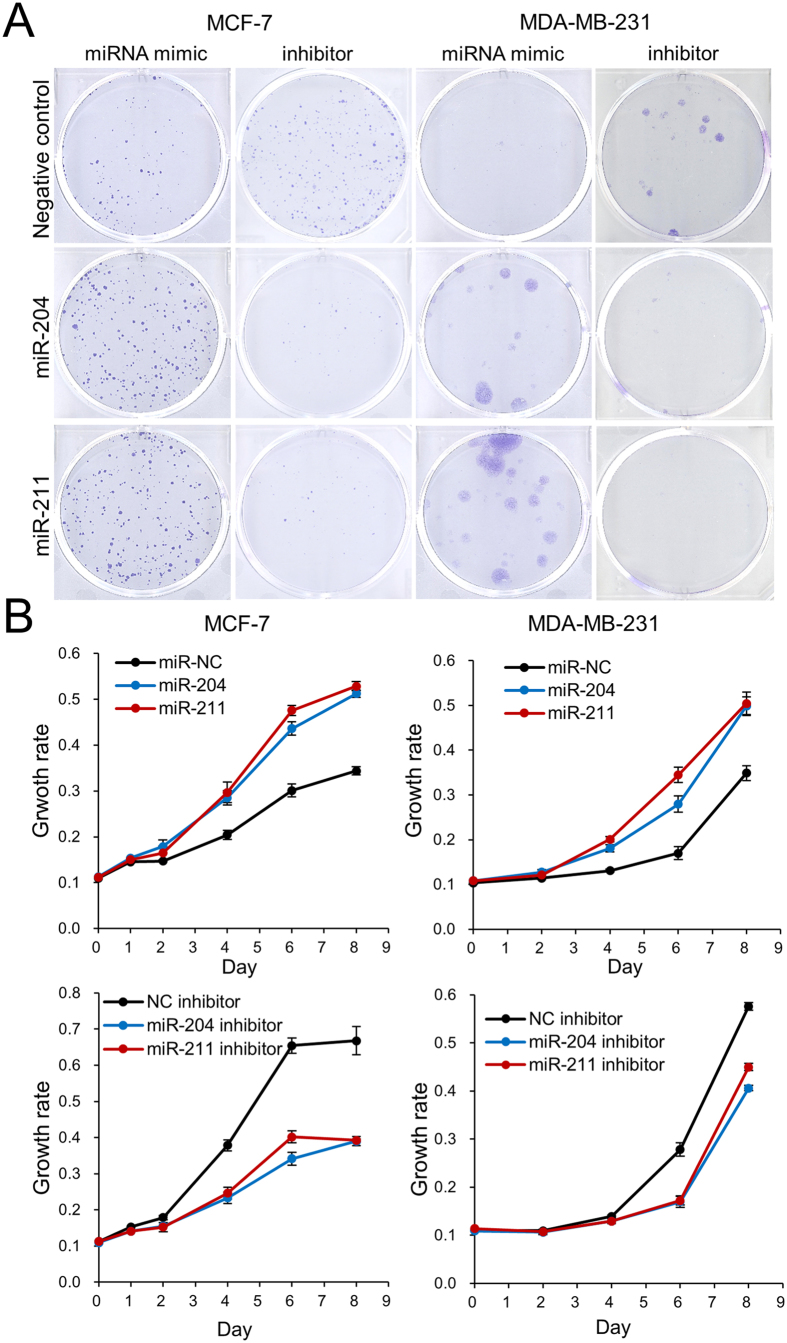
miR-204/211 induce cell proliferation of MCF-7 and MDA-MB-231. Mimic miR or inhibitor miR of miR-204/211 was transiently transfected into the breast cancer cells to examine their effect on cell proliferation. After transfection, colony formation analysis (**A**) and CCK-8 assay (**B**) were performed. The colony images are taken from at least three independent experiments, and the cell proliferation is shown as means with standard errors.

Based on the information obtained from the microarray analysis that suggested the possible cell-proliferative activity of miR-204/211, the effect of the miRs on cellular proliferation was examined in the MCF-10A, MCF-7, and MDA-MB-231 cell after ectopically inducing up- and down-regulation of the miRs. The cell activity change was monitored in two experimental ways: colony formation assay and cell proliferation assay. When miR mimics were transfected into the MCF-7 cell, they increased the number as well as size of the cultured colony compared to a mock mimic (Fig. 5A). In contrast, inhibitor-based downregulation of the miRs resulted in a decrease of the colony number as well as size (Supplementary Fig. S1 and Fig. 5A). Next, cell proliferation was monitored using a dye-based method. A higher growth rate was observed when mimic miR was transfected in both cases of miR-204/211 compared to that of a control where a mock mimic RNA was used (Fig. 5B). Meanwhile, a lower growth rate was shown when the miRs were inhibited by an inhibitor miR (Fig. 5B).

**Figure 6:**
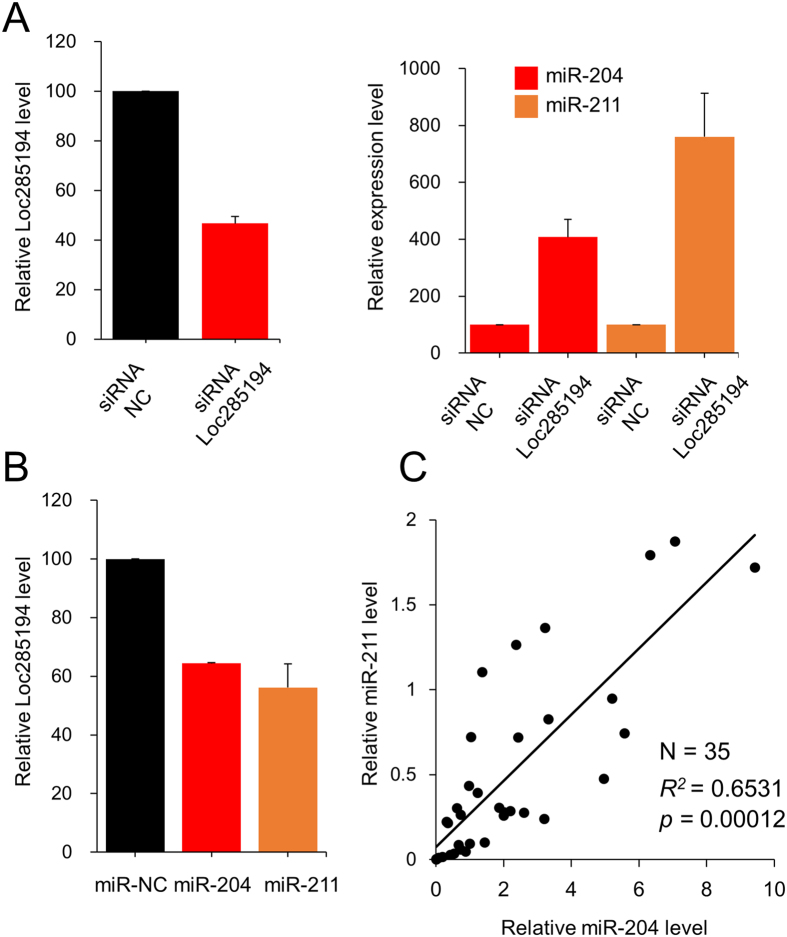
Association of expression between miR-204 and miR-211. (**A**) Reciprocal regulation between miR-204/211 and loc285194. Loc285914 was downregulated in MCF-7 using siRNA and expression of miR-204/211 was determined by RT-PCR. (**B**) Mimic of miR-204 or miR-211 was transfected into MCF-7 (Supplementary Fig. S1) and expression of loc285914 was determined by RT-PCR. Three independent experiments were performed, and the expression level is shown as means with standard errors. (**C**) Association of miR-204 and miR-211 expression in breast cancer tissue. Expression of miR-204/211 was examined by RT-PCR in 35 breast cancer tissues and normalized with the value of U6 RNA. Coefficient of determination (*R*^*2*^) was calculated by linear regression.

In the case of MDA-MB-231 (Fig. 5) and MCF-10A (Supplementary Fig. S4), the results for the colony formation as well as cell proliferation assays showed a similar pattern to those of MCF-7, although the level of change was not the same, indicating a similar manner of the two miRs to change the degree of colony formation and cell proliferation in the three cell types. Recently, a tumor suppressor long non-coding RNA (lncRNA), loc285194, was found to downregulate miR-211 and to be reciprocally downregulated by miR-211 in colon cancer^31^. We also examined the relationship between loc285194 and miR-204/211 in breast cancer cells. When loc285194 was downregulated, the expression of miR-211 was increased in MCF-7 (Fig. 6A). Reciprocally, when the miR was upregulated using mimic, the expression of the loc285194 was decreased (Fig. 6B). MiR-204 showed a similar reciprocal regulation, although the changed level is less than that of miR-211. In a clinical experiment, we examined the association between miR-204 and miR-211 expression in 35 breast cancer tissues. We found a high *R*-value (*R*^*2*^ = 0.6531), supporting the similar expression pattern of the two miRs (Fig. 6C). Taken together, these results indicate that the two miRs act similarly in breast cancer cells to drive proliferation and to suppress cell death.

### MiR-204/211 downregulate tumor suppressor genes

**Figure 7:**
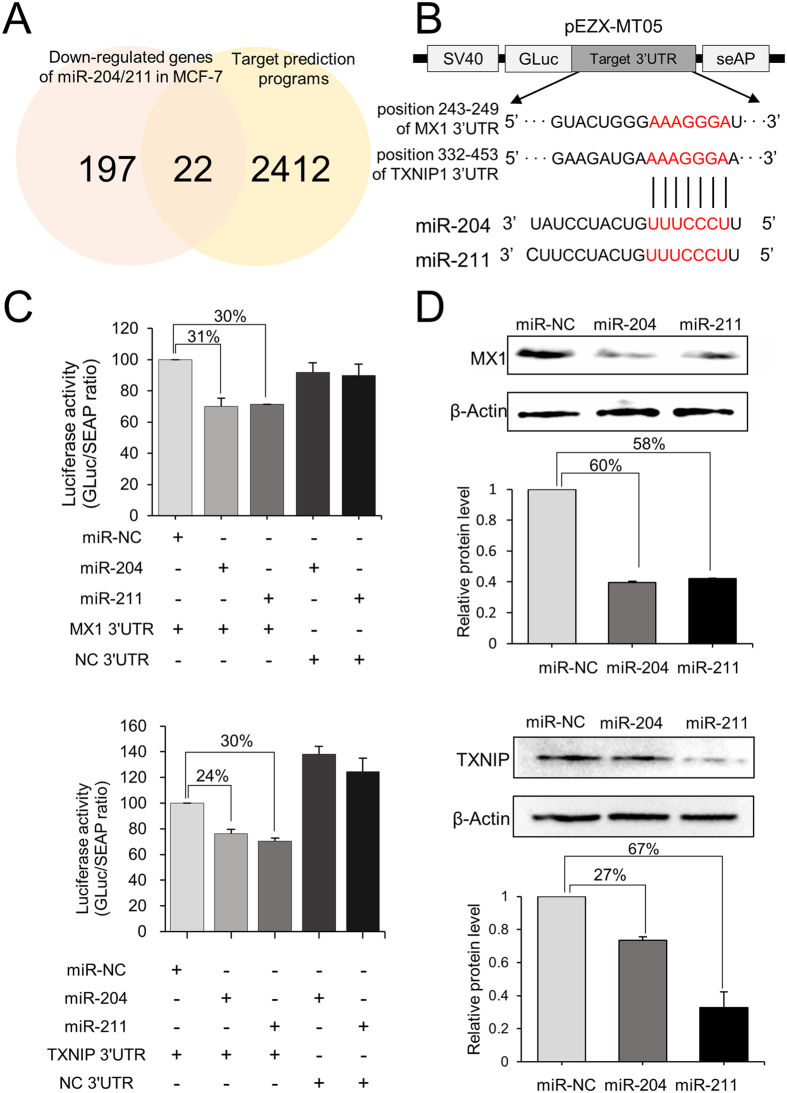
MiR-204/miR-211 directly target and downregulate MX1 and TXNIP. (**A**) Prediction of common target genes for miR-204/211. Target genes were screened from expression array data (|fold change| ≥1.5) and six public databases (miRanda, miRWalk, PITA5, TargetScan, DIANAmT, and RNA22). Genes that hit at least five databases were selected. (**B**) Schematic diagram of the luciferase vector containing the 3′-UTR of MX1 and TXNIP. The plausible binding sequence between miRs and target genes is indicated in red. (**C**) Luciferase assay for MCF-7 cells co-transfected with various combinations of recombinant luciferase vector with miR or NC (negative control). (**D**) Western blot analysis of MX1 and TXNIP after overexpressing miR-204/211 in MCF-7. The band intensity was measured by Image Lab software and indicated as bar graphs.

To examine whether miR-204/211 directly regulate their target genes by binding to the 3′-untranslated region (3′-UTR), potential target genes were first selected, which commonly appeared in the 197 genes that were downregulated by the two miRs in MCF-7 and in the 2412 genes that were revealed as potential targets by *in silico* approaches. As a result, 22 genes were represented (Fig. 7A and Supplementary Table S1) and genes such as SERP1^33^ that has been already proved to be a direct target of the miRs were excluded, resulting in six genes. Luciferase analysis of the six genes eventually identified MX1 and TXNIP to be novel targets. MX1 is an interferon-inducible GTPase, and it has been known to inhibit tumor cell motility and invasion^34^. TXNIP is a potent negative regulator of glucose uptake, aerobic glycolysis, and glycolytic gene expression; thus, its repression is associated with active tumor growth^35^,^36^. When the 3′-UTR containing the target sequence was placed downstream of the luciferase gene (Fig. 7B), the luciferase expression decreased by up to 24–31% by both miRs compared to the control miR in the HEK-293T cells (Fig. 7C). Moreover, overexpression of either miR in the MCF-7 cells decreased expression of the protein, as judged by Western blot analysis (Fig. 7D). These results demonstrate the oncogenic role of miR-204/211 by directly suppressing tumor suppressors. Interrogating expression of MX1 and TXNIP using the GOBO database revealed that patients with higher expression were more likely to have a higher rate of distant metastasis-free survival (DMSF) (*p* < 0.05) (Supplementary Fig. S5).

## Discussion

This study was carried out to reveal the role of miR-204/211 in breast cancer cells by identifying target genes at a genome-wide level. After integrating the interaction network and examining cellular activity, a few novel roles of the miRs involved in the tumorigenesis of cancer cells were elucidated. Previous studies indicate differential roles of miR-204/211 in various cancer types. miR-204 acts as a tumor suppressor in pancreatic^37^, gastric^38^, and breast cancer^28^. Accordingly, oncogenes such as MCL-1, RAB22A, and JAK2 have been revealed as targets of miR-204. MiR-204 was downregulated in MCF-7 and MDA-MB-231 than in MCF-10A and overexpression of the miR resulted in increase of apoptosis^28^. On the other hand, the miR shows a dual activity in prostate cancer by acting as a tumor suppressor in adenocarcinoma cells and as an oncomiR in neuroendocrine-like cancer^21^. In the case of miR-211, it is difficult to specify its activity, because it acts as a tumor suppressor in hepatocellular carcinoma^39^, ovarian cancer^40^, and melanoma^41^ but as an oncogene in colorectal^20^, head and neck^42^, and oral carcinoma^43^. In colorectal and head and neck carcinoma, miR-211 is upregulated and thereby induces cell proliferation. Meanwhile, miR-211 is significantly downregulated in breast cancer^44^. Re-expression of miR-211 suppressed cell growth, cell cycle, migration, and invasion in the triple-negative breast cancer cell line MDA-MB-231, demonstrating its suppressor activity.

The most prominent feature of the two miRs revealed in this study is that they tend to drive cells toward being oncogenic. This fact was supported by a series of molecular and cellular experimental evidences. First, miR-204/211 targeted many of the same tumor suppressors. For example, XAF1^45^, HIPK2^46^, and RARRES3^47^,^48^, which were downregulated by miR-204/211, are already known to be tumor suppressors. In addition, we identified two novel target genes of miR-204/211, MX1 and TXNIP, which are tumor suppressors. Activity loss of MX1 stimulated tumor development in prostate cancer^49^ and so did TXNIP in various types of cancer^36^. Second, the significant diseases such as “cancer” or “apoptosis” constructed by the genes dysregulated by the miRs were biased to favor cell proliferation as well as inhibit cell death. Third, at the cell level, up- or down-regulation of the miRs in the MCF-7 and MDA-MB-231 cells indicated pro-proliferative activity, which was judged by colony formation and dye-based proliferation assays.

Another characteristic of miR-204/211 found in this study was that both miRs behaved in a similar (oncogenic) way in the two breast cancer cell lines (MCF-7 and MDA-MB-231). When different miRs share the same seed sequence, they target the same 3′-UTR sequence in some cases. For example, miR-30b and miR-30d, which share the same seven bases (GUAAACA), both target GALNT7 in melanoma^50^. However, miR-96 and miR-182 regulated different targets even though they had the same seed sequence. It has been shown that sequences flanking the seed sequence are also essential to recognize and regulate the target sites through interacting with components of RISC^51^. It is speculated that other factors cooperate with miRs to determine the scope of target genes and pathways in a given cell. The expression of a specific target protein can be fine-tuned by alternative cleavage and polyadenylation to the corresponding RNA^52^. Approximately one-third of the analyzable human miR recognition elements (MREs) in MiRTarBase and TarBase can potentially perform splicing-regulated fine-tuning.

Our results of miR-204 appear inconsistent, at least in the case of MDA-MB-231, with those of Wang *et al.*^28^ that suggest a tumor-suppressive role of miR-204 in the cell. These opposite activities may be due to the possible dual activity of miR-204 as it behaves in prostate cancer. Based on our results and previous studies, miR-204/211 seem to exhibit dual activity depending on the specific status of the breast cancer cell. The molecular mechanism of this opposite activity is yet to be explored. Further studies on the upper regulatory molecules of the miRs could reveal how their activities are split into either tumor suppressors or oncogenes. Currently, TrkB is known to regulate miR-204 in endometrial cancer cells^53^.

Taken together, miR-204/miR-211 share a group of target genes, many of which are tumor suppressors. This characteristic drives the breast cancer cells toward being oncogenic. These findings could contribute to the development of cancer treatment options by modulating the miRs themselves and their target genes.

## Materials and Methods

### Cell culture and transfection

Human breast cell lines (MCF-10A (normal), MCF-7 (cancer), and MDA-MB-231 (cancer)) and a human embryonic kidney cell line (HEK-293T) were purchased from American Type Culture Collection (ATCC; Manassas, VA, USA). MCF-10A was cultured in MEBM basal medium (Lonza, Basel, Switzerland) supplemented with the MEGM SingleQuot Kit and cholera toxin. MCF-7 and MDA-MB-231 were grown in RPMI-1640 (Gibco, Los Angeles, CA, USA) and HEK-293T was cultured in DMEM (Gibco) with 10% fetal bovine serum and 1% penicillin/streptomycin. All cell lines were maintained in a humidified environment with 5% CO_2_ at 37 °C. MiR mimics, control miRs, inhibitor miRs, and siRNA were synthesized from Bioneer (Korea) (Supplementary Table S2), and their sequences were based on the miRBase sequence database (http://www.mirbase.org/). All miRs, inhibitors, and siRNAs were transiently transfected into cells of 50% confluency at 30 nM of final concentration using Lipofectamine RNAiMAX (Invitrogen, Carlsbad, CA, USA) diluted in Opti-MEM I Medium (Gibco). Cells were harvested for purification of RNA and protein 24–48 h after transfection.

### Study subjects

All patients provided written informed consent to donate removed tissue to the National Cancer Center (NCC) in Korea and samples were obtained according to protocols approved by the Research Ethics Board of NCC. Thirty-five breast cancers (BrCa) tissues were obtained from patients who had undergone surgery between 2013 and 2014 at NCC.

### RNA isolation and real-time qRT-PCR

Total RNA from miR-transfected cells and tissues was extracted using the miRNeasy Mini Kit (Qiagen, Valencia, CA, USA) according to the manufacturer’s protocol, and the extracted RNA was reverse-transcribed using the miScript II RT Kit (Qiagen). The expression levels of miR-204/211 were quantified by real-time quantitative RT-PCR using the miScript SYBR Green PCR Kit (Qiagen) and miScript Primer Assays as the primers, and then normalized to U6. To quantify loc285194 expression, RT-PCR was performed using KAPA SYBR FAST qPCR Kit Master Mix ABI Prism (Kapa Biosystems, Wilmington, MA, USA) and normalized to GAPDH. Each sample was assayed in triplicate and calculated according to the 2^−ΔΔCt^ method. All RT-PCRs were conducted on an ABI 7300 instrument (Applied Biosystems, Foster City, CA, USA).

### Genome-wide expression array

Two micrograms of total RNA from miR- or control miR-transfected cells were used to obtain genome-wide gene expression profiles. Biotin-labeled cDNA was synthesized and applied to the Illumina HumanHT-12 v4 Expression Beadchip (Illumina, San Diego, CA, USA) that covers 47,000 human targets. Microarray data analysis was carried out by Illumina GenomeStudio v2011.1 (Gene Expression Module v1.9.0). The microarray data were uploaded to the Gene Expression Omnibus (GEO) database, and they can be accessed through its website (http://www.ncbi.nlm.nih.gov/geo/) under the series accession number GSE75292.

### Pathway and clustering analysis

IPA software (Ingenuity Systems, Redwood City, CA) was utilized to construct biologically significant canonical pathways and regulatory interaction networks from the genes differentially expressed (|fold change| ≥1.5, p-value < 0.05) by the mimic miR in MCF-10A and MCF-7. The network name was adopted from the internal Ingenuity Pathway Analysis annotation. The highest confidence functional network was designated as the top network. Clustering analysis was performed and visualized using Cluster 3.0 software (http://bonsai.hgc.jp/~mdehoon/software/cluster/) and the TreeView v1.1.6 program (http://jtreeview.sourceforge.net/).

### Cell proliferation assay

The cell proliferation assay was performed using Cell Counting Kit-8 (Dojindo, Japan). In 96-well plates, 3 × 10^3^ cells/well of MCF-7 and MDA-MB-231 were seeded in triplicate. After seeding, miR-204, miR-211, their inhibitors, and corresponding negative controls were transfected using Lipofectamine RNAiMAX (Invitrogen) and incubated for 0, 24, 48, and 96 h in a humidified incubator (37 °C, 5% CO_2_), and 10 μl of CCK-8 solution was added into each well. Two h after incubation, the absorbance value was measured at 450 nm using a microplate reader, and the absorbance value at 600 nm was subtracted from the A450 value to eliminate the turbidity effect.

### Colony formation assay

After seeding 5 × 10^3^ MCF-7 and MDA-MB-231 cells in each well of 6-well plates, 30 nM miR-204 mimic, miR-211 mimic, miR-negative control, and their inhibitors were transiently transfected using Lipofectamine RNAiMAX (Invitrogen). The cells were cultured for 14 days, being refreshed with fresh medium of the same composition every 3 days. Colonies were then rinsed with 1×PBS (Gibco), fixed (methanol:acetic acid = 7:1), and stained with 0.5% crystal violet solution. After incubation for 1 h at room temperature, the staining solution was removed, and the colonies were washed and air-dried.

### Luciferase reporter assay

For the luciferase reporter assay, a full-length 3′-UTR of miR target genes was subcloned downstream of the Gaussia luciferase (GLuc) reporter gene of the pEZX-MT05 vector (GeneCopoeia, Rockville, MD, USA). Then, 24 h after seeding HEK-293T cells in 24-well plates, 30 nM miR-204, miR-211, or miR-negative control were co-transfected with 150 ng of the recombinant plasmid using Lipofectamine 3000 and PLUS reagent (Invitrogen). Two days after the transfections, luciferase activity was detected with a Secrete-Pair Dual Luminescence Assay Kit (GeneCopoeia) according to the manufacturer’s protocol. The transfection efficiency variation was eliminated by normalizing the GLuc expression over SEAP activities.

### Western blot analysis

Proteins were extracted from miR-204-, miR-211-, or miR-negative control-transfected MCF-7 cells using Pierce IP Lysis Buffer (Thermo Fisher Scientific, Waltham, MA, USA) with a protease inhibitor cocktail (Thermo Fisher Scientific). Diluted protein samples were separated on 4–12% gradient SDS-PAGE gels (Koma Biotech, Korea) and transferred to polyvinylidene difluoride membranes (Whatman, UK). Then, the membranes were blocked in 5% non-fat powdered milk (Bio Basic, Canada) for 1 h and incubated for 1 h with anti-MX1 (1:500, Santa Cruz, Dallas, Texas, USA) and anti-TXNIP (1:500, Novus, St. Charles, MO, USA) antibodies. The membranes were then rinsed in 0.01% Tween-20 TBS five times for 5 min each and incubated with horseradish peroxidase conjugated secondary donkey anti-rabbit antibody (1:5000, Amersham, UK) for 1 h. The bands were detected by an EzWay ECL Western Blot Substrate Kit (Koma Biotech) and Image Lab software (Bio-Rad, Hercules, CA, USA). As an endogenous reference, rabbit polyclonal anti-β-actin antibody (1:500) (Novus) was used.

### Data mining and statistical analysis

The gene expression-based outcome for breast cancer online (GOBO) tool (http://co.bmc.lu.se/gobo) is designed for prognostic validation of genes in a pooled breast cancer data set comprising 1,881 cases from 11 public microarray data sets^54^. It was used to predict prognosis of breast cancer patients according to the expression level of MX1 and TXNIP. In the expression chip data, genes with adjusted *p*-values equal to or greater than 0.05 were removed from further analysis. Filtered genes were defined as differentially expressed if they displayed a 1.5 fold or higher difference in expression levels between mimic and control miR-transfected cells to enrich for biologically relevant expression changes. In pathway analyses, *p*-values for individual networks that indicated the likelihood of obtaining the same number of transcripts or greater in a random set were obtained using a Fisher’s exact test. Linear regression was conducted to calculate the coefficient of determination (*R*^*2*^) and the statistical significance of the correlation. Student’s *t*-test was used to detect differences in the expression level between the mimic and control miR and between inhibitor and control miR using SPSS for Windows, release 17.0 (SPSS, Chicago, IL, USA). *p*-values < 0.05 were considered statistically significant.

## Additional Information

**How to cite this article**: Lee, H. *et al.* Genome-wide identification of target genes for miR-204 and miR-211 identifies their proliferation stimulatory role in breast cancer cells. *Sci. Rep.* **6**, 25287; doi: 10.1038/srep25287 (2016).

## Supplementary Material

Supplementary Information
