# Supplementary material for: Genome-wide identification of target genes for miR-204 and miR-211 identifies their proliferation stimulatory role in breast cancer cells
Source: Sci Rep. 2016 Apr 28;6:25287. doi: 10.1038/srep25287 (PMC4848534; doi:10.1038/srep25287)

Genome-wide identification of target genes for miR-204 and miR-211 identifies their proliferation stimulatory role in breast cancer cells

Hyunkyung Lee[1,†], Seungyeon Lee[1,†], Hansol Bae[1], Han-Sung Kang[2], and Sun Jung Kim[1]

[1]Department of Life Science, Dongguk University-Seoul, Goyang, Republic of Korea

[2]Research Institute and Hospital, National Cancer Center, Goyang, Republic of Korea

Supporting Information

Table S1. Potential target genes of miR-204/211*

Gene symbol	Accession	Description	Fold change miR-204	Fold change miR-211
IFIT2	NM_001547.4	Interferon-Induced Protein With Tetratricopeptide Repeats 2	-26.2	-33.9
MX1	NM_002462.2	MX Dynamin-Like GTPase 1	-14.7	-21.6
SAMD9	NM_017654.2	Sterile Alpha Motif Domain Containing 9	-13.7	-17.5
LAMP3	NM_014398.2	Lysosomal-Associated Membrane Protein 3	-9.1	-10.4
DDX60L	NM_001012967.1	DEAD (Asp-Glu-Ala-Asp) Box Polypeptide 60-Like	-7.1	-7.3
IFI44	NM_006417.3	Interferon-Induced Protein 44	-7.0	-5.2
TRANK1	NM_014831.1	TPR and ankyrin repeat-containing protein 1	-5.8	-5.6
SP100	NM_001080391.1	SP100 Nuclear Antigen	-3.4	-3.8
NCOA7	NM_181782.2	Nuclear Receptor Coactivator 7	-3.1	-2.6
TRIM38	NM_006355.2	Tripartite Motif Containing 38	-2.8	-2.7
IFIT5	NM_012420.1	Interferon-Induced Protein With Tetratricopeptide Repeats 5	-2.4	-2.3
PML	XM_942288.1	Promyelocytic Leukemia	-2.2	-2.3
ADAR	NM_001111.3	Adenosine Deaminase, RNA-Specific	-2.1	-2.2
MOB3C	NM_145279.4	MOB kinase activator 3C	-2.1	-1.8
ADAR	NM_015840.2	Adenosine Deaminase, RNA-Specific	-2.1	-2.2
SERP1	NM_014445.3	Stress-Associated Endoplasmic Reticulum Protein 1	-1.9	-1.5
LAMP3	NM_014398.2	Lysosomal-Associated Membrane Protein 3	-1.9	-1.9
PSME1	NM_176783.1	Proteasome (Prosome, Macropain) Activator Subunit 1 (PA28 Alpha)	-1.8	-1.6
ZFYVE26	NM_015346.2	Zinc Finger, FYVE Domain Containing 26	-1.7	-1.7
GREB1	NM_033090.2	Growth Regulation By Estrogen In Breast Cancer 1	-1.7	-1.6
TXNIP	NM_006472.2	Thioredoxin Interacting Protein	-1.5	-2.0
PSME1	NM_006263.2	Proteasome (Prosome, Macropain) Activator Subunit 1 (PA28 Alpha	-1.5	-1.5

*Target genes were screened from expression array data (|fold change|≥1.5) and six public databases (miRanda, miRWalk, PITA5, TargetScan, DIANAmT, and RNA22).

Table S2. Sequence information for mimics, inhibitors, siRNAs, and control

		Catalog no.	Product name or sequence (5'-3')	Supplier
**primer**	miR-204-5p	MS00003773	Hs_miR-204_1 miScript Primer Assay	Qiagen
	miR-211-5p	MS00003808	Hs_miR-211_1 miScript Primer Assay	Qiagen
	RNU6	MS00033740	Hs_RNU6-2_11 miScript Primer Assay	Qiagen
	LOC285194		F: TGTGCCTGTTTGACCTCTGA	Genotech
			R: AGGAAGGATAAAAGACCGACCA	Genotech
**miRNA mimic**	miR-204-5p		UUCCCUUUGUCAUCCUAUGCCU	Bioneer
	miR-211-5p		UUCCCUUUGUCAUCCUUCGCCU	Bioneer
	control miRNA	SMC-3001	miRNA mimic Negative control #2	Bioneer
**miRNA inhibitor**	miR-204-5p		AGGCAUAGGAUGACAAAGGGAA	Bioneer
	miR-211-5p		AGGCGAAGGAUGACAAAGGGAA	Bioneer
	control	SMC-2101	miRNA inhibitor Negative control #1	Bioneer
**siRNA**	LOC285194		Sense : GGCCAAACCCUCAAUGAAUtt	Bioneer
			Antisense: AUUCAUUGAGGGUUUGGCCtg	
	control	SN-1003	Negative control siRNA	Bioneer

Figure S1. Transfection of mimics and inhibitors for miR-204/211 into MCF-10A and MCF-7. Mimics (A) and inhibitors (B) for each miR were transiently transfected into the indicated cell and expression was examined by real-time RT-PCR. NC: negative control mimic or inhibitor. All samples were performed at least three times and the result is shown as average with standard errors.

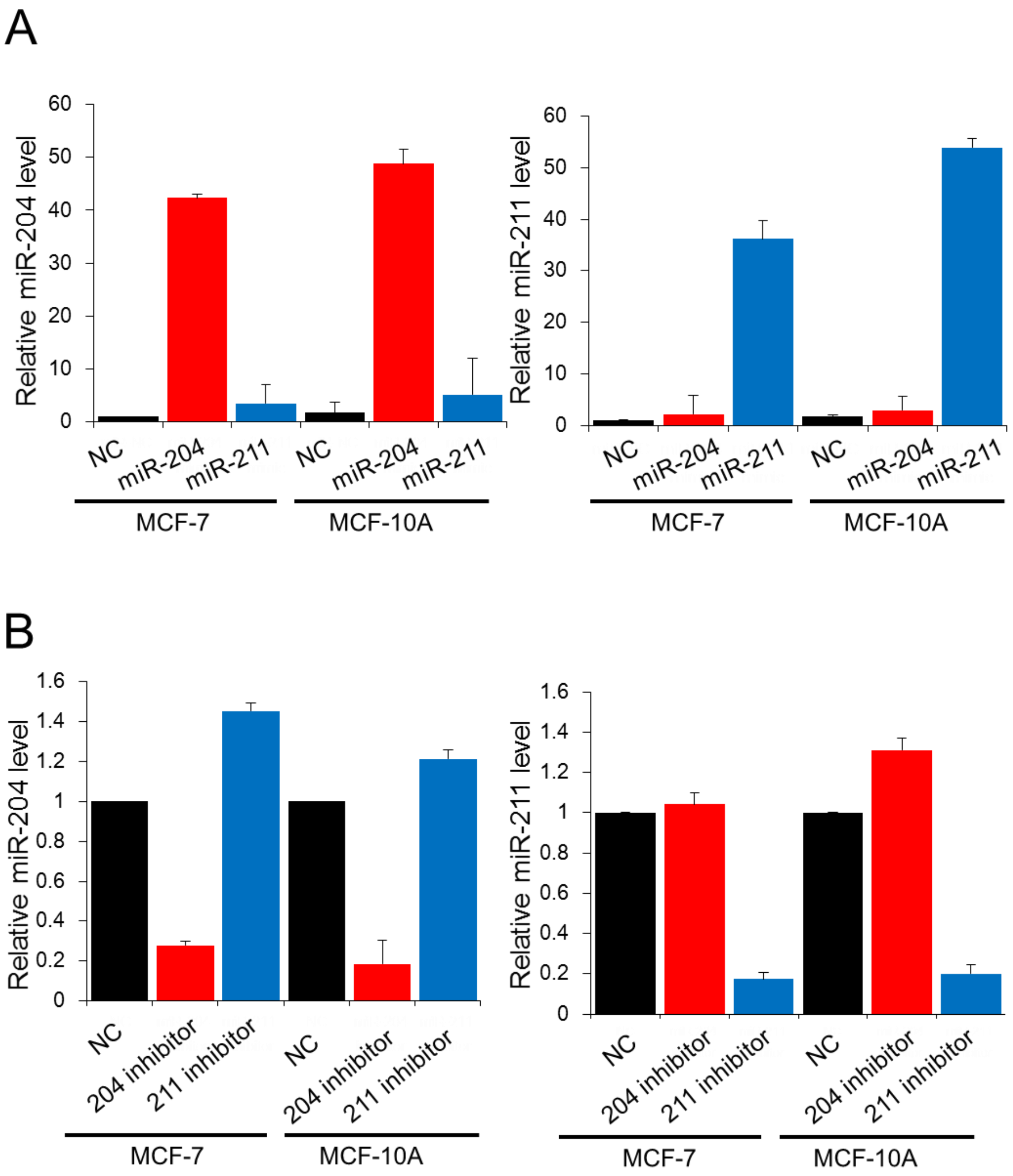

Figure S2. Highest confidence network of genes displaying altered expression by miR-204/211 in MCF-10A. The highest confidence network was constructed using IPA from 145 and 162 dysregulated genes by miR-204 (A) and miR-211 (B), respectively. The top network in MCF-10A for miR-204/211 was "Cancer, Cellular Movement, Organismal Injury, and Abnormalities."

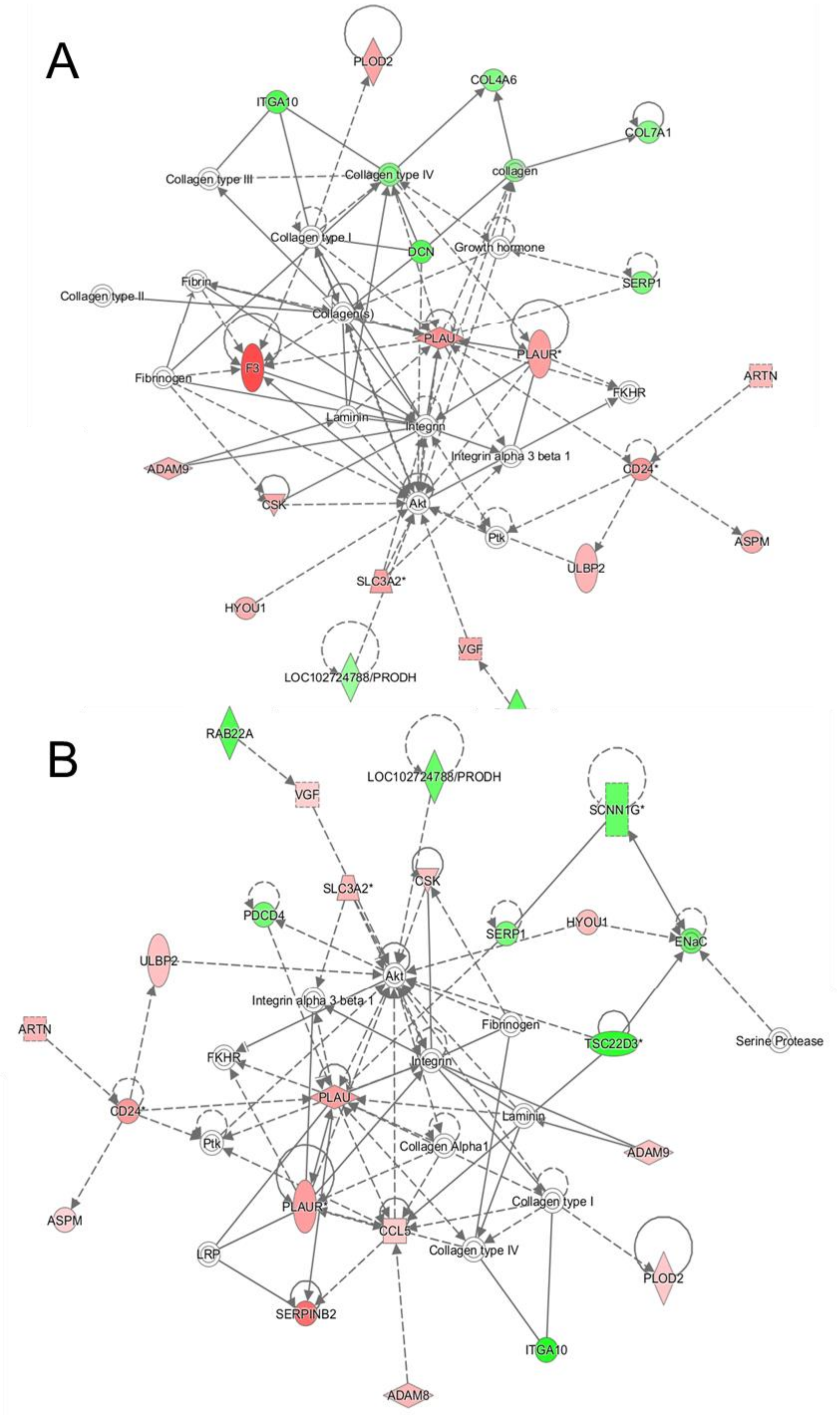

Figure S3. Pathways most strongly associated with the genes significantly dysregulated by miR-204/211 in MCF-10A. The top 10 functional categories are given for altered genes by miR-204 (A) and miR-211 (B) overexpressed in MCF-7 cells. In both miRs, "Regulation of cytokine production in macrophage and T helper cells" appeared as the top pathway having the lowest *p*-value.

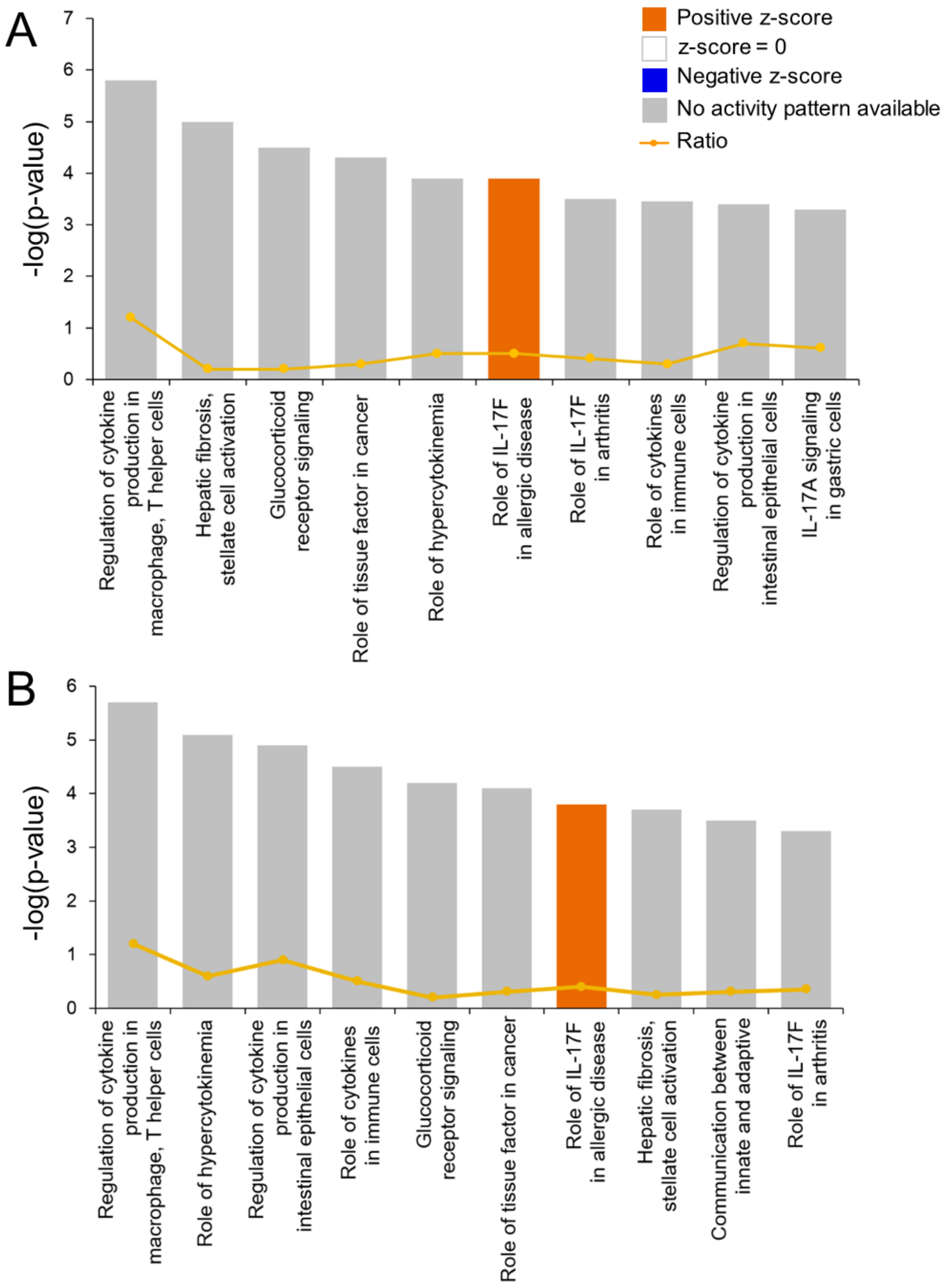

Figure S4. miR-204/211 induce cell proliferation of MCF-10A. Mimic miR or inhibitor miR of miR-204/211 was transiently transfected into MCF-10A to examine their effect on cell proliferation. After transfection, colony formation analysis (A) and CCK-8 assay (B) were performed. The colony images are taken from at least three independent experiments, and the cell proliferation is shown as means with standard errors.

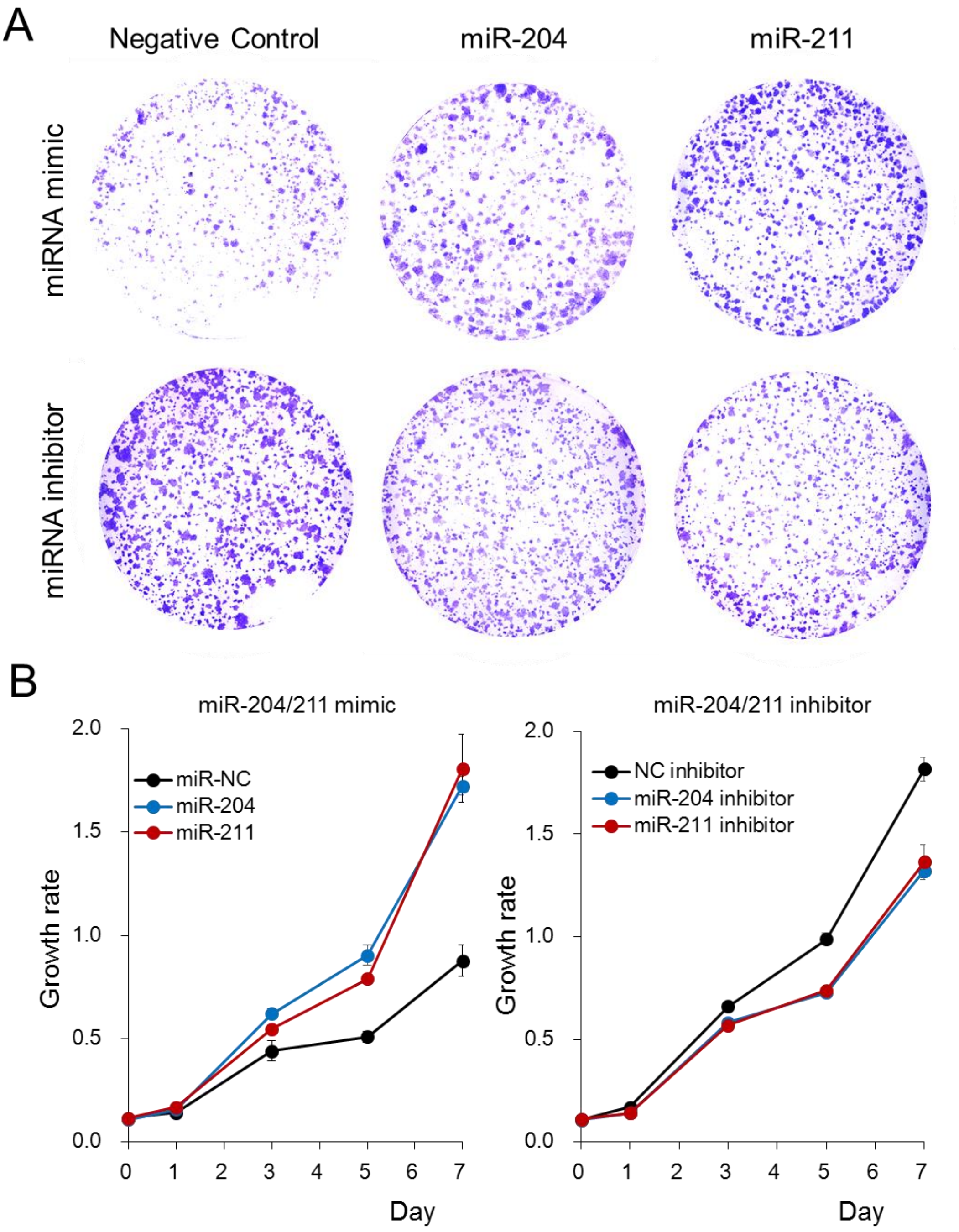

Figure S5. Kaplan-Meier survival analysis of MX1 and TXNIP expression in the breast cancer patients. Samples were stratified into tertiles based on MX1 and TXNIP expression level. The log-rank test was performed in tumor samples using distant metastasis-free survival (DMFS) as the endpoint. High MX1 **(A)** and TXNIP **(B)** expression is significantly associated with higher DMFS over time among HER2-enriched tumors (n=166) and all tumors (n = 1,379), respectively.

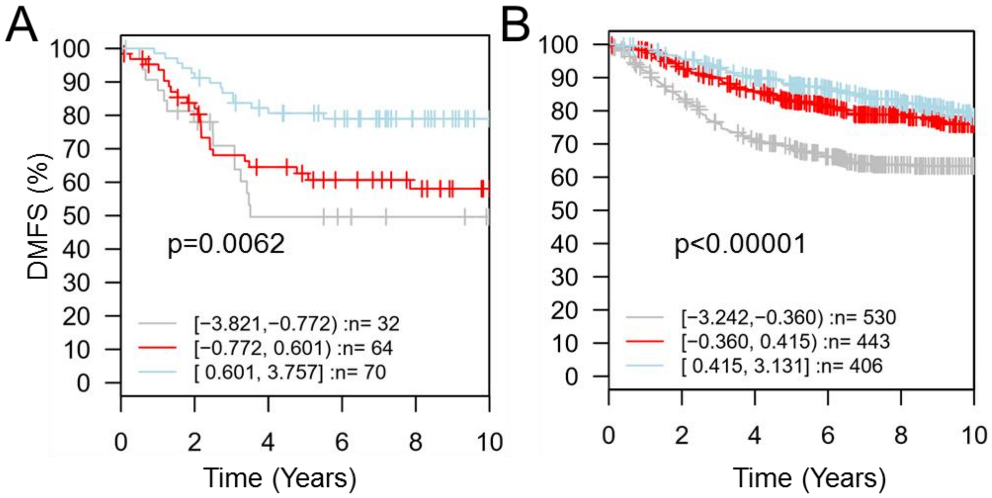

Supplement: Supplementary Information [file srep25287-s1.pdf]
